# Effect of low bicarbonate substitution solution on CO_2_ removal rate in the combined system of extracorporeal CO_2_ removal and continuous renal replacement therapy

**DOI:** 10.1186/s40635-025-00827-8

**Published:** 2025-11-19

**Authors:** Zhicheng Qian, Rui Zhang, Yuxuan Wang, Hao He, Shike Geng, Yang Li, Xueyan Yuan, Yi Yang, Haibo Qiu, Songqiao Liu, Ling Liu

**Affiliations:** 1 Jiangsu Provincial Key Laboratory of Critical Care Medicine, Department of Critical Care Medicine, Zhongda Hospital, School of Medicine, Southeast University, Nanjing, China; 2 Department of Critical Care Medicine, Beijing Anzhen Nanchong Hospital, Capital Medical University & Nanchong Central Hospital, Nanchong, Sichuan China; 3 Department of Emergency, Shandong First Medical University & Shandong Academy of Medical Sciences, Central Hospital Affiliated to Shandong First Medical University, Jinan, China; 4 Department of Critical Care Medicine, West China Hospital/West China School of Medicine, Sichuan University, Chengdu, Sichuan China; 5 The First People’s Hospital of Lianyungang, The Affiliated Lianyungang Hospital of Xuzhou Medical University, Lianyungang, 222000 Jiangsu China

**Keywords:** Extracorporeal carbon dioxide removal, Continuous veno-venous hemofiltration, Low bicarbonate substitution solution, Carbon dioxide removal rate, Hypercapnia

## Abstract

**Background:**

The concurrent application of extracorporeal carbon dioxide removal (ECCO₂R) and continuous renal replacement therapy (CRRT) delivers essential respiratory and renal support. However, the use of bicarbonate (HCO₃⁻) in substitution solution increases the external HCO₃⁻ load and affect the carbon dioxide removal rate (VCO₂). This study aims to investigate the influence of low bicarbonate substitution solution on VCO₂ within the combined ECCO₂R-CRRT system.

**Methods:**

This crossover study was conducted with hypercapnic pigs and patients with acute respiratory distress syndrome (ARDS). In pigs, we tested two extracorporeal blood flow rates (200 and 350 mL/min) alongside three continuous veno-venous hemofiltration (CVVH) strategies: a control group receiving ECCO₂R alone without CVVH, a low HCO₃⁻ group receiving ECCO₂R combined with CVVH (HCO₃⁻ concentration of 16 mmol/L at a substitution rate of 30 mL/kg/h), and a normal HCO₃⁻ group (HCO₃⁻ concentration of 25 mmol/L). Respiratory variables, hemodynamic parameters, and VCO₂ were measured 30 min after each intervention. In ARDS patients, we also assessed ECCO₂R combined with these CVVH strategies. The primary endpoint was the comparison of VCO₂ among the three groups in both the pig and patient.

**Results:**

This study involved 12 hypercapnic pigs. At a blood flow rate of 200 mL/min, the VCO_2_ were significantly different among groups (*P* = 0.029). The VCO₂ in the low HCO₃⁻ group (51.7 ± 6.0 mL/min) was significantly higher than that in the normal HCO₃⁻ group (46.1 ± 2.9 mL/min) and comparable to the control group (50.3 ± 5.4 mL/min). However, at a blood flow rate of 350 mL/min, VCO₂ values were similar across all three groups. In 10 ARDS patients with a mean age of 64 ± 8 years, the PaCO₂ was 60.0 ± 4.7 mmHg prior to ECCO₂R. At a blood flow rate of 293 ± 59 mL/min, VCO₂ did not change significantly in the low HCO₃⁻ group (77.0 ± 16.2 mL/min) compared to the control group (75.2 ± 15.9 mL/min), a decrease was noted in the normal HCO₃⁻ group (69.9 ± 16.6 mL/min, *P* < 0.010).

**Conclusion:**

A low bicarbonate concentration of 16 mmol/L in the substitution solution may optimize CO₂ elimination in the ECCO₂R–CRRT system, especially at lower extracorporeal blood flow rates.

**Supplementary Information:**

The online version contains supplementary material available at 10.1186/s40635-025-00827-8.

## Introduction

Extracorporeal carbon dioxide removal (ECCO₂R) is a technique that provides artificial respiratory support by removing carbon dioxide (CO₂) from the blood through an extracorporeal gas exchanger [1]. This advanced method, which utilizes a low-flow blood circuit, has emerged as a promising adjunctive therapy over the past few decades, particularly for patients with acute respiratory distress syndrome (ARDS) and acute exacerbations of chronic obstructive pulmonary disease (COPD) [2–5]. The main goal of ECCO_2_R in ARDS is to facilitate lung protective ventilation, while avoiding hypercapnic acidosis.

Approximately 40% of patients with ARDS develop acute kidney injury (AKI) and require continuous renal replacement therapy (CRRT) [6]. As a result, researchers have explored the feasibility and safety of combining ECCO₂R with CRRT to provide simultaneous respiratory and renal support [2, 7, 8]. However, previous studies have indicated that the combination of ECCO₂R and CRRT typically achieves a limited carbon dioxide removal rate (VCO₂) of no more than 50 mL/min [2, 8]. CO₂ can be eliminated as both gas and bicarbonate through the ECCO₂R-CRRT system. Furthermore, low bicarbonate (HCO₃⁻) concentrations in the CRRT circuit may enhance CO₂ clearance, as indicated by mathematical models and in vitro dialysis experiments [9]. This inspires us that HCO₃⁻ concentrations in CRRT circuit may impact the CO₂ clearance capability, especially when the efficiency of membrane lung varied by clinical factors. Higher or normal bicarbonate levels in the pre-dilution solution may increase CO_2_ load prior to the membrane lung, thereby reducing the overall system efficiency. Based on this, we hypothesize that lower HCO₃⁻ concentrations in the substitution solution could be more effective in facilitating CO_2_ clearance, provided acceptable acid–base balance was maintained, when ECCO_2_R is combined with CRRT in clinical practice.

This study aims to assess the effect of low HCO₃⁻ substitution solution VCO₂ in the combined ECCO₂R-CRRT system using hypercapnic pigs and patients with ARDS.

## Methods

**Fig. 1:**
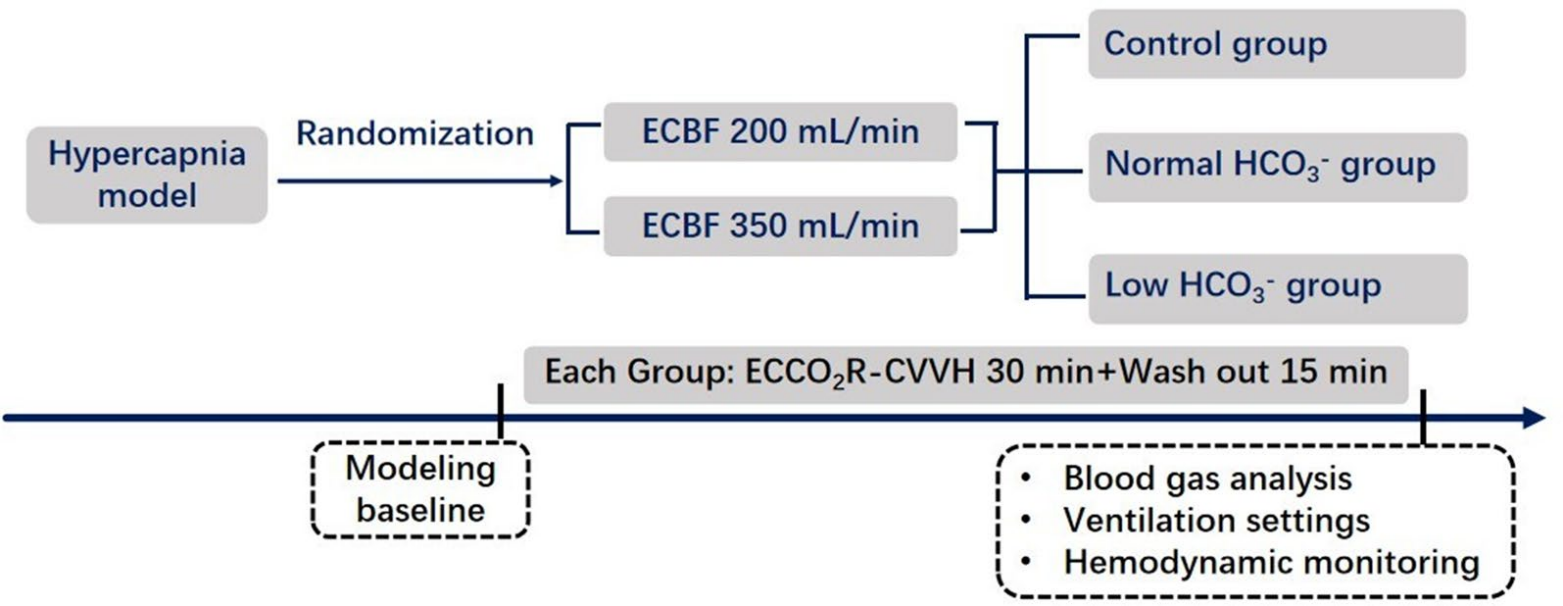
Schematic representation of the preclinical study protocol. Control group is ECCO_2_R alone; normal HCO_3_^−^ group receiving ECCO_2_R combined with CVVH (HCO_3_^−^ concentration of 25 mmol/L at a substitution rate of 30 ml.kg^−1^.h^−1^); low HCO_3_^−^ group with HCO_3_^−^ concentration of 16 mmol/L. Extracorporeal circuit and arterial blood gas analysis were obtained at the end of each group. ECBF, extracorporeal blood flow

We conducted two series of ethics-approved, physiological, randomized, crossover studies in hypercapnic pigs and ARDS patients. Figure 1 outlines the protocol for preclinical study, and detailed methods are provided in the Supplement.

### Series 1: Hypercapnic pigs

The study was approved by the Animal Ethics Committee of Southeast University, China (Approval number: 20210901045). Detailed methodologies are outlined in the Supplementary Material. Briefly, the ECCO₂R-CRRT system was performed on 12 healthy domestic pigs (mean weight 56.5 ± 4.9 kg; 6 males and 6 females). All animals were cared for in accordance with the hospital's standards for the care and use of laboratory animals.

### Animal preparation

Twelve healthy domestic pigs were anesthetized, paralyzed, and intubated in the supine position. Mechanical ventilation (Servo-I, Maquet, Sweden) was initiated in volume-controlled mode, with a fraction of inspired oxygen (FiO₂) of 100%, a tidal volume of 6 mL/kg, a respiratory rate of 15 breaths/min, and positive end-expiratory pressure (PEEP) of 5 cmH₂O. The animals were instrumented with central venous and femoral artery catheters. An electrolyte solution was infused at 2–3 mL/kg/h throughout the experiment. Colloid solution and norepinephrine were administered to maintain the mean arterial pressure (MAP) above 60 mmHg when necessary.

### Extracorporeal circuit

**Fig. 2:**
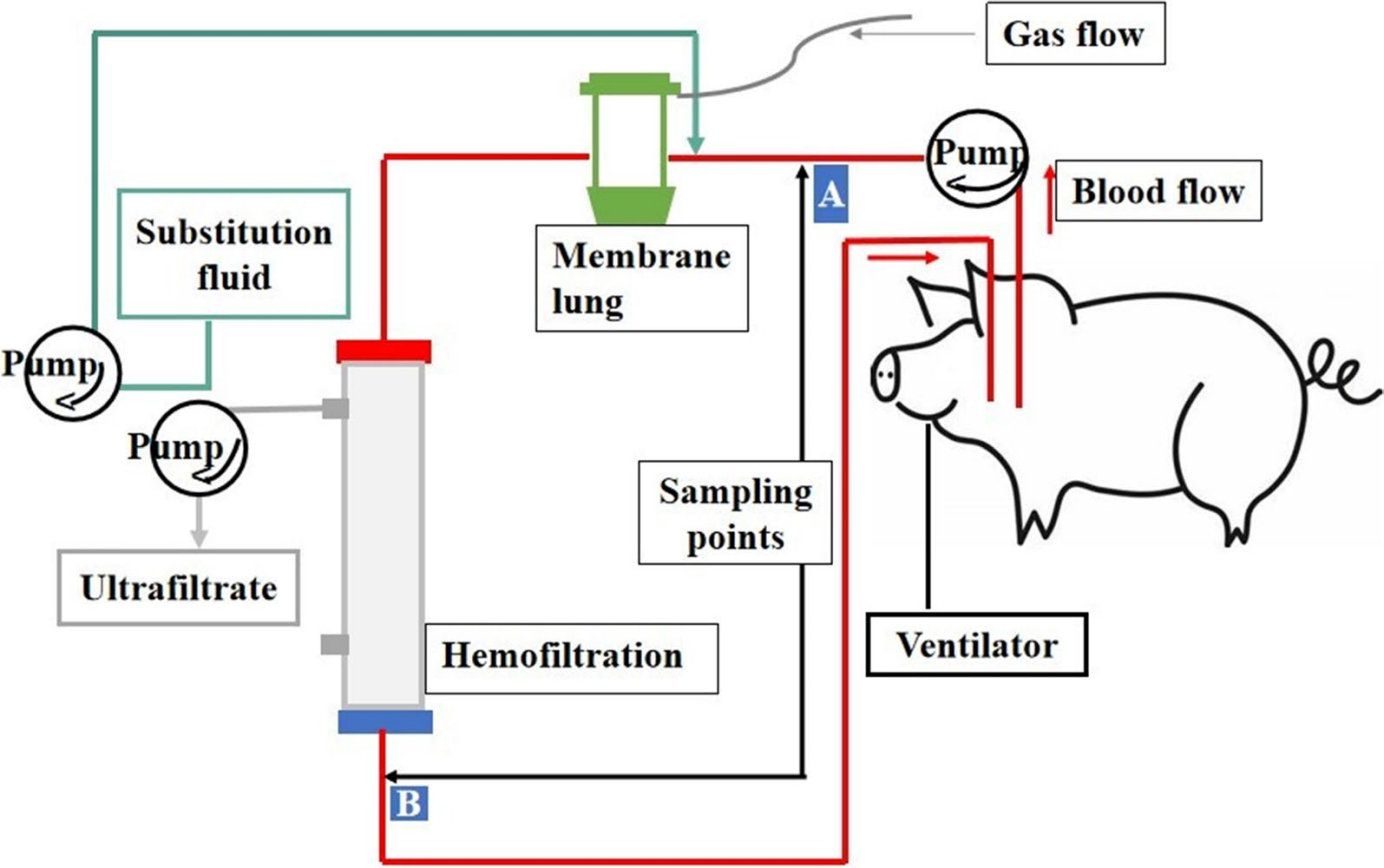
Diagram of the extracorporeal CO2 removal plus continuous renal replacement therapy experiment. Sampling sites (**A**) and (**B**) represent inflow and post-filter blood draw sites, respectively

A 14F double-lumen dialysis catheter (Arrowg + ard Blue^®^, ARROW, USA) was inserted into the jugular vein. The ECCO₂R-CRRT system included a membrane lung (OMNIset®, BBraun, Germany) with a total surface area of 1.81 m^2^ and a hemoperfusion filter (OMNIfilter®, BBraun, Germany) with a surface area of 1.6 m^2^. The filling volume of the ECCO₂R and CRRT was 187 mL and 94 mL, respectively. The membrane lung was connected in series with the hemofilter, positioned upstream of the hemofilter. A schematic representation of the integrated system is provided in Fig. 2.

### Experimental design

Hypercapnia (target PaCO₂ of 70–89 mmHg) was induced by reducing the respiratory rate as previously described [10] (details are given in the Supplement). After stabilizing hypercapnia for 15 min, we initiated ECCO₂R combined with continuous veno-venous hemofiltration (CVVH) The sweep gas flow rate was kept constant at 10 L/min. To achieve target bicarbonate concentrations of 25 mmol/L (normal HCO₃⁻ group) and 16 mmol/L (low HCO₃⁻ group), 5% sodium bicarbonate was aseptically added to the basic substitution solution (Shijiazhuang Fourth Pharmaceutical Company, China). Detailed compositions of the substitution solution are provided in Tables S1 and S2. The effluent flow rate of CVVH was set at 30 mL/kg/h with 100% pre-dilution. Post-dilution and dialysis were not used. The ultrafiltration rate was adjusted to match the infusion rate of the substitution solution, ensuring no net fluid loss during CVVH.

In this study, two levels of extracorporeal blood flow (ECBF) rates (200 and 350 mL/min) were combined with three CVVH strategies: a control group undergoing ECCO₂R without CVVH, a low HCO₃⁻ group receiving ECCO₂R with CVVH at a bicarbonate concentration of 16 mmol/L, and a normal bicarbonate group with a concentration of 25 mmol/L, applied at a substitution rate of 30 mL/kg/h. These combinations resulted in six distinct therapeutic steps, applied in a randomized order, each maintained for 30 min. To prevent carry-over effects, both ECCO₂R and CVVH were paused for a 15-min washout period between steps. If necessary, adjustments to the respiratory rate were made to maintain PaCO₂ within the target range of 70 to 89 mmHg prior to initiating the next step. Ventilator settings were kept constant during each therapeutic step.

### Measurements and outcomes

Before initiating the sweep gas flow and at the conclusion of each therapeutic step, data were recorded, including blood gas analysis, ventilatory parameters, and hemodynamic variables. Blood samples for gas analysis were obtained from the femoral artery catheter, as well as two sites on the extracorporeal circuit (pre-membrane lung site and post-hemofilter site), which were used to calculate the CO₂ removal rate (VCO₂). (see Fig. 2).

The primary endpoint as the CO₂ removal rate (VCO₂), calculated using the following formula [2, 11]:

VCO₂ (mL/min) = (CtCO₂-PRE − CtCO₂-POST) × 22.4 × ECBF (mL/min) / 1000, where CtCO₂ (mmol/L) = (0.0307 × PCO₂) + actual HCO₃⁻, and CtCO₂-PRE and CtCO₂-POST represent the CO₂ content at the pre- and post-membrane lung sites, respectively.

To minimize variability due to inlet PCO₂, VCO₂ was normalized to a reference value of 45 mmHg [12]: VCO₂ (normalized) = VCO₂ (calculated) × 45 / PCO₂-PRE.

Secondary outcomes included the change in arterial CO₂ tension (ΔPaCO₂) and pH (ΔpH) before and after each intervention. Heart rate, mean arterial pressure (MAP) and cardiac output (CO) were also measured.

### Series 2: Clinical study

#### Participants

The study was registered on ClinicalTrials.gov (NCT05989971) and approved by the Institutional Ethics Committee of Zhongda Hospital (Approval number: 2023ZDSYLL091-P01). Written informed consent was obtained from the participants or their legally authorized representatives. The study was conducted in the intensive care unit of Zhongda Hospital, Southeast University, China, from April 2023 to March 2024, with the recommendations of the Helsinki Declaration.

Inclusion criteria were as follows: (1) age between 18 and 85 years; (2) using Extracorporeal carbon dioxide removal combined with continuous renal replacement therapy. Indications for initiating ECCO_2_R therapy in patients with ARDS: Vt ≤ 6 ml/kg PBW, and Pplat(plateau pressure) > 25 cmH_2_O, PaCO_2_ > 55 mmHg, pH < 7.30, PaO_2_/FiO_2_ ≥ 100 mmHg [13]. Exclusion criteria included: (1) severe hemodynamic instability (increased dosage of vasoactive drugs or MAP ≤ 65 mmHg within two hours); (2) severe electrolyte deficiency (including severe hyponatremia (blood sodium < 130 mmol/L) and hypokalemia (blood potassium < 3.0 mmol/L)); (3) participate in other intervention studies within 30 days; and (4) do not consent this research.

### Interventions

In this study, we employed the ECCO₂R-CRRT system (OMNIfilter^®^, BBraun, Germany), using 100% oxygen as the sweep gas. Three therapeutic steps were applied in a randomized sequence: (1) a control group undergoing ECCO₂R without CVVH, (2) a low HCO₃⁻ group receiving ECCO₂R with CVVH at a bicarbonate concentration of 16 mmol/L, and (3) a normal HCO₃⁻ group with a bicarbonate concentration of 25 mmol/L, both administered at a substitution rate of 30 mL/kg/h with 100% pre-dilution. Each intervention lasted for 30 min, without a washout period between steps, to prevent repeated increases in PaCO₂.

Systemic anticoagulation with heparin was employed to prevent clotting in the circuit, targeting an activated partial thromboplastin time of 60 to 80 s. The ECBF was set to the maximum value that ensured stable blood flow, while the sweep gas flow was maintained at a constant rate of 10 L/min throughout the study. Initial ventilator settings and adjustments to ventilator parameters were determined by the attending physician. Further details regarding the clinical protocol can be found in the Supplement.

### Measurements and outcomes

Data collection procedures and outcome definitions were aligned with those utilized in the animal study. Adverse events and clinical outcomes were also documented (see Supplement).

## Statistical analysis

Categorical variables were presented as counts (percentages), and continuous variables were expressed as mean ± standard deviation (SD) or median with interquartile range (IQR), as appropriate. Comparisons among the control and treatment groups were performed using repeated measures analysis of variance (ANOVA), with Bonferroni correction applied for pairwise comparisons. In cases where the assumption of sphericity was violated, the Kruskal–Wallis test was employed, followed by post hoc pairwise comparisons using Dunn’s correction.

All statistical analyses were performed using STATA version 17.0 (StataCorp, College Station, TX, USA) and GraphPad Prism version 9.0 (GraphPad Software, San Diego, CA, USA). A two-sided *P*-value of < 0.05 was considered statistically significant.

## Results

### Series 1: Hypercapnic pigs

Following ventilator adjustments, hypercapnia was induced in all 12 animals, resulting in a PaCO₂ of 78.3 ± 6.8 mmHg prior to ECCO₂R initiation at an ECBF of 200 mL/min, and 78.7 ± 7.5 mmHg at 350 mL/min (Table 1). Baseline PaCO₂ levels were restored after each washout period before advancing to the subsequent therapeutic step (Table 1).

**Table 1:** Variations of respiratory parameters among extracorporeal CO_2_ removal strategies in hypercapnic pigs (n = 12)

Variables	Before modeling	Control group			Normal HCO_3_^−^group			Low HCO_3_^−^group		
		Baseline	After removal	*P* value	Baseline	After removal	*P* value	Baseline	After removal	*P* value
Blood gas analysis (ECBF of 200 mL/min)										
Arterial pH	7.44 ± 0.05	7.21 ± 0.07	7.26 ± 0.05	< 0.001	7.21 ± 0.06	7.25 ± 0.05	< 0.001	7.20 ± 0.06	7.28 ± 0.06	< 0.001
PaCO_2_ (mmHg)	42.7 ± 7.0	78.3 ± 6.8	69.8 ± 8.4	< 0.001	79.1 ± 5.7	73.6 ± 7.2	0.007	78.3 ± 6.3	69.4 ± 8.8	< 0.001
PaO_2_/FiO_2_ (mmHg)	458.4 ± 56.3	347.3 ± 33.0	364.8 ± 39.2	0.28	347.8 ± 45.3	367.3 ± 52.5	0.01	408.9 ± 107.3	374.2 ± 64.3	0.18
HCO_3_^−^ (mmol/L)	28.2 ± 4.0	31.9 ± 2.8	31.3 ± 3.0	0.166	31.7 ± 3.2	31.5 ± 3.4	0.59	33.2 ± 3.2	32.4 ± 3.2	0.233
MV (L/min)	5.28 ± 0.33	1.30 ± 0.51	1.30 ± 0.51	–	1.30 ± 0.51	1.30 ± 0.51	–	1.30 ± 0.51	1.30 ± 0.51	–
Blood gas analysis (ECBF of 350 mL/min)										
Arterial pH	7.44 ± 0.05	7.22 ± 0.06	7.30 ± 0.08	< 0.001	7.23 ± 0.05	7.31 ± 0.06	< 0.001	7.21 ± 0.05	7.28 ± 0.04	< 0.001
PaCO_2_ (mmHg)	42.7 ± 7.0	78.7 ± 7.5	64.8 ± 9.5	0.001	76.3 ± 4.8	63.7 ± 8.1	< 0.001	78.0 ± 4.1	64.8 ± 5.4	< 0.001
PaO_2_/FiO_2_ (mmHg)	458.4 ± 56.3	396.9 ± 67.9	423.4 ± 85.2	0.13	402.0 ± 74.6	419.4 ± 84.5	0.353	343.0 ± 55.7	364.4 ± 60.2	0.175
HCO_3_^−^ (mmol/L)	28.2 ± 4.0	32.1 ± 3.0	31.2 ± 2.6	0.001	31.8 ± 2.8	30.9 ± 2.8	0.002	31.4 ± 2.4	30.1 ± 2.5	< 0.001
MV (L/min)	5.28 ± 0.33	1.36 ± 0.50	1.36 ± 0.50	–	1.36 ± 0.50	1.36 ± 0.50	–	1.36 ± 0.50	1.36 ± 0.50	–

*ECBF* extracorporeal blood flow. *PaCO*_*2*_ partial pressure of arterial carbon dioxide; *PaO*_*2*_ partial pressure of arterial oxygen; *FiO*_*2*_ fraction of inspired oxygen; *MV* Minute ventilation;

*P* value denotes comparisons between baseline and after removal at the same treatment strategy

**Fig. 3:**
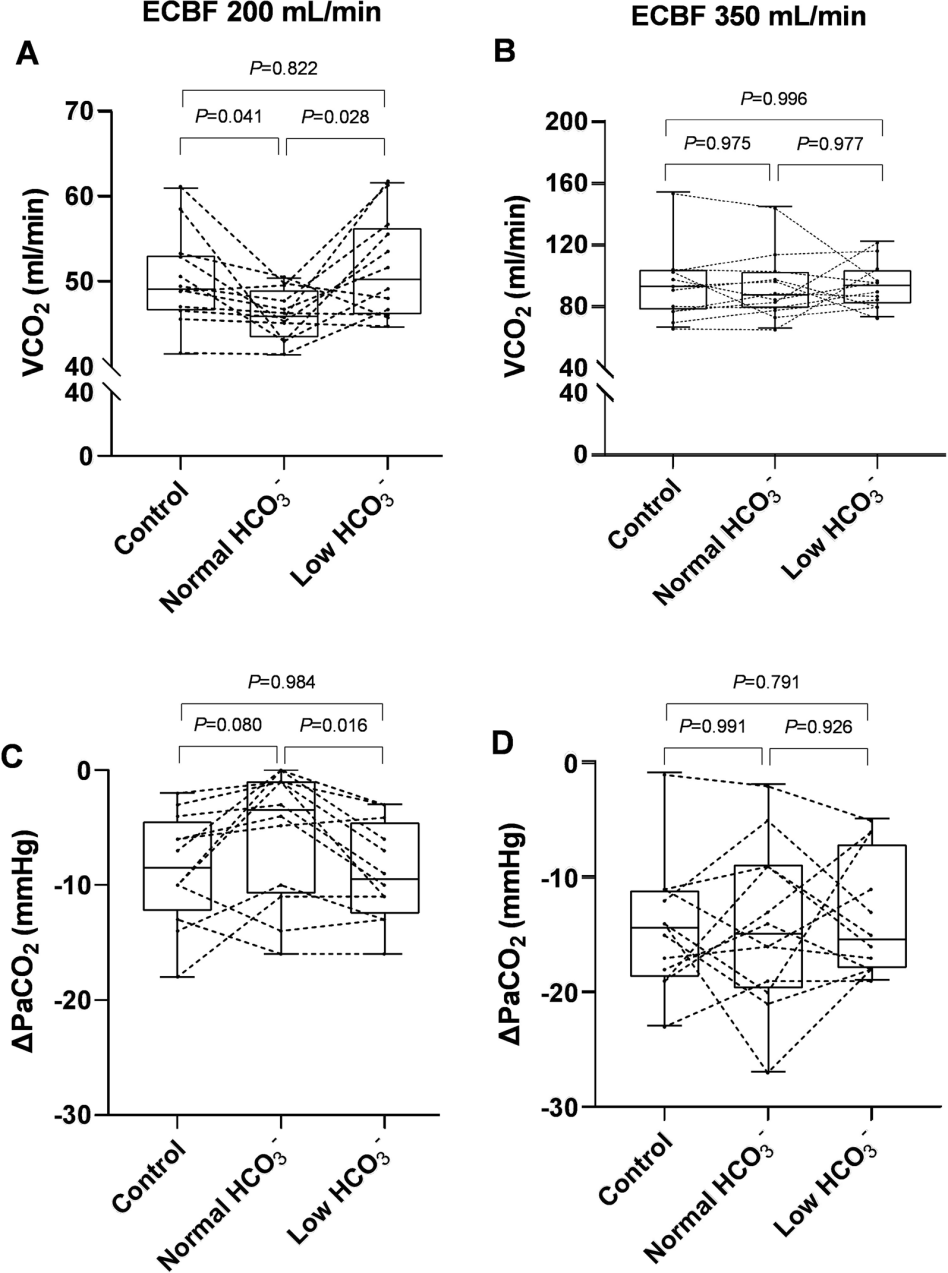
The VCO_2_ and ΔPaCO_2_ of three groups in pigs at ECBF of 200 mL/min and 350 mL/min, Individual pig data are represented by black dots. ECBF, extracorporeal blood flow; VCO_2_, extracorporeal carbon dioxide removal rate; ΔPaCO_2_ = PaCO_2_ after removal $-$ PaCO_2_ before removal

The VCO₂ values across the six therapeutic steps are showed in Fig. 3. At an ECBF of 200 mL/min, significant differences were observed among the groups (*P* = 0.029). The VCO₂ in the low HCO₃⁻ group (51.7 ± 6.0 mL/min) was significantly higher than that in the normal HCO₃⁻ group (46.1 ± 2.9 mL/min) and similar to the control group (50.3 ± 5.4 mL/min) (Fig. 3A). In contrast, at an ECBF of 350 mL/min, no significant differences in VCO₂ were detected among the groups (Fig. 3B).

In each group, PaCO₂ decreased and arterial pH increased significantly following ECCO₂R at both ECBF levels of 200 mL/min and 350 mL/min. However, there were no significant differences in PaO₂/FiO₂ ratios among groups (Table 1). At an ECBF of 200 mL/min, significant differences in ΔPaCO₂ were observed among the groups (*P* = 0.038). The low HCO₃⁻ group experienced a greater reduction in PaCO₂ compared to the normal HCO₃⁻ group (-8.8 ± 4.3 mmHg vs -5.5 ± 5.7 mmHg, *P* = 0.016) (Fig. 3C), however, there were no significant differences in ΔpH between groups (0.05 ± 0.03 vs 0.04 ± 0.03, *P* = 0.347). At an ECBF of 350 mL/min, ΔPaCO₂ and Δheart rate (HR) values were comparable across groups, although HR were significantly lower after ECCO₂R within each group. Additionally, mean arterial pressure (MAP), and cardiac output (CO) remained unchanged following ECCO₂R for all therapeutic steps (Table S3).

### Series 2: Clinical study

Ten patients with ARDS were enrolled in the study, with a median age of 64 ± 8 years, and all completed the treatment protocol. Detailed ECCO₂R-CRRT settings are presented in Table 2. Prior to the initiation of ECCO₂R, the PaCO₂ and PaO₂/FiO₂ were 60.0 ± 4.7 mmHg and 173.4 ± 48.2 mmHg, respectively (Table 3).

**Table 2:** The ECCO₂R-CRRT settings and outcome of ARDS patients (n = 10)

No	Sex	Age (years)	Cause of ARDS	Catheter position	Catheter ID (Fr)	ECBF (mL/min)	GF (L/min)	Duration of ECCO_2_R (day)	Adverse event	Outcome
1	Female	58	Pneumonia	Jugular	14	300	10	3	None	Death
2	Female	50	Pneumonia	femoral	14	350	10	3	None	Death
3	Female	70	COVID-19	Jugular	14	350	10	2	None	Survival
4	Male	61	COVID-19	Jugular	14	220	10	2	None	Survival
5	Male	77	COVID-19	femoral	12	180	10	3	None	Survival
6	Female	67	COVID-19	femoral	12	250	10	13	None	Death
7	Female	71	Pneumonia	Jugular	14	300	10	2	Intracranial hemorrhage	Death
8	Male	61	Pneumonia	Jugular	14	330	10	23	None	Survival
9	Female	64	Pneumonia	femoral	14	300	10	6	None	Death
10	Female	60	Pneumonia	Jugular	14	350	10	3	None	Death
Summary	3Male/7Female	64 ± 8	6Pneumonia/4COVID-19	6 Jugular/4 femoral	2 12Fr/8 14Fr	293±59	10	3 [2, 8]	1 Intracranial hemorrhage	4 Survival/6 Death

*ARDS* acute respiratory distress syndrome, *ECBF* Extracorporeal blood flow, *GF* gas flow

**Table 3:** The variables of baseline and different extracorporeal CO_2_ removal strategies in patients (n = 10)

Variables	Baseline	Control group	Normal HCO_3_^−^group	Low HCO_3_^−^group	*P* value
Blood gas analysis					
Arterial pH	7.24 ± 0.03	7.39 ± 0.07^*^	7.41 ± 0.06^*^	7.43 ± 0.04^*^	0.127
PaCO_2_ (mmHg)	60.0 ± 4.7	48.6 ± 6.9^*^	48.1 ± 5.4^*^	46.1 ± 3.5^*^	0.176
HCO_3_^−^ (mmol/L)	32.7 ± 3.3	32.1 ± 5.2	31.6 ± 4.4	29.1 ± 4.8^*^	0.058
PaO_2_/FiO_2_ (mmHg)	173.4 ± 48.2	171.5 ± 55.4	188.7 ± 91.7	196.2 ± 82.6	0.188
Ventilatory parameters					
Tidal volume (ml/kg, PBW)	5.2 ± 0.8	4.6 ± 0.7^*^	4.6 ± 0.7^*^	4.5 ± 0.6^*^	0.274
Respiratory rate (breaths/min)	30.5 ± 3.1	30.5 ± 3.1	30.5 ± 3.1	30.5 ± 3.1	-
MV (L/min)	9.1 ± 1.7	8.0 ± 1.4^*^	7.9 ± 1.4^*^	7.8 ± 1.4^*^	0.282
Pplat (cmH_2_O)	28.0 ± 2.0	24.7 ± 3.1^*^	24.1 ± 3.8^*^	23.2 ± 3.2^*^	0.14
Driving pressure (cmH_2_O)	19.5 ± 2.4	16.5 ± 3.4^*^	16.6 ± 3.3^*^	15.7 ± 3.2^*^	0.123
Hemodynamics					
Heart rate (beats/min)	97 ± 19	89 ± 13^*^	91 ± 11^*^	88 ± 10^*^	0.463
MAP (mmHg)	71 ± 12	65 ± 5	70 ± 6	72 ± 4	0.818

*PaCO2* partial pressure of arterial carbon dioxide, *PaO2* partial pressure of arterial oxygen, *FiO2* fraction of inspired oxygen, *PBW* predicted body weight, *MV* minute ventilation; *Pplat* plateau pressure. *MAP* mean arterial pressure. P value denote comparisons among three groups. *P < 0.05, compared with baseline

**Fig. 4:**
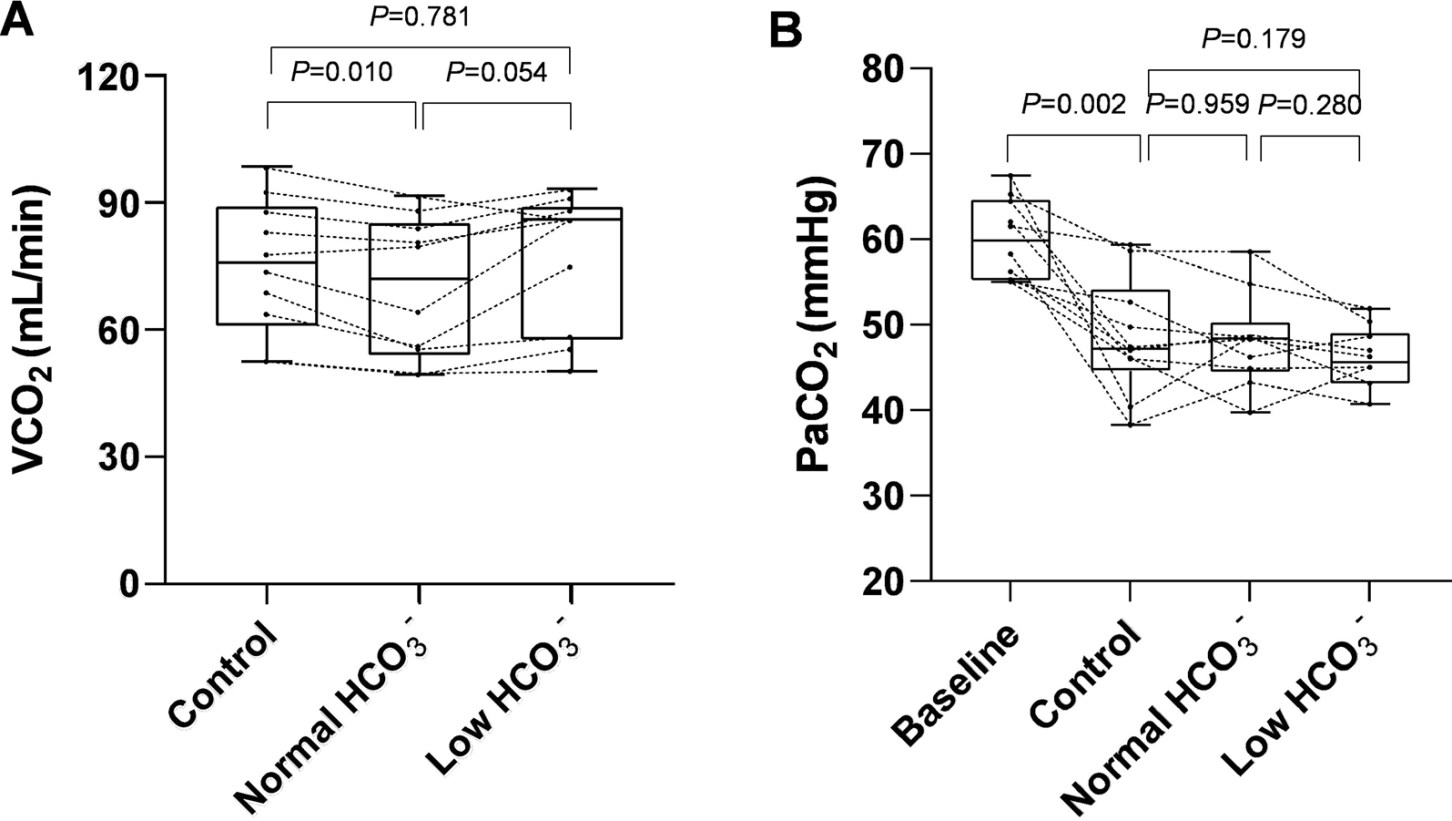
The VCO_2_ and PaCO_2_ of different extracorporeal CO_2_ removal strategies in patients, Individual patient data are represented by black dots. VCO_2_, extracorporeal carbon dioxide removal rate

The VCO₂ values across the three therapeutic steps are presented in Fig. 4. At a blood flow rate of 293 ± 59 mL/min, significant differences in VCO₂ levels were observed among groups (*P* = 0.033). While VCO₂ did not change significantly in the low HCO₃⁻ group compared to the control group, a decrease was noted in the normal HCO₃⁻ group (*P* < 0.050) (Fig. 4A).

Following the initiation of ECCO₂R, respiratory acidosis improved, demonstrated by a decrease in PaCO₂ and an increase in arterial pH (Table 3). The PaCO_2_ in the low HCO₃⁻ group was comparable to the normal HCO₃⁻ group (46.1 ± 3.5 vs 48.1 ± 5.4 mmHg, *P* = 0.280) (Fig. 4B). After ECCO₂R, tidal volume decreased from 5.2 ± 0.8 ml/kg predicted body weight to 4.5 ± 0.6 ml/kg predicted body weight, and plateau pressure decreased from 28.0 ± 2.0 cmH₂O to 23.2 ± 3.2 cmH₂O. However, the respiratory rate did not change after ECCO₂R (Table 3). ECCO_2_R was maintained for 3 [2, 8] days. One SAEs (intracranial hemorrhage) were considered attributable to ECCO_2_R-CRRT. A total of 4/10 patients (40%) were alive at hospital discharge.

## Discussion

This study demonstrates that in hypercapnic animals and ARDS patients, using an ECCO₂R-CRRT system with a normal bicarbonate concentration of 25 mmol/L in the substitution solution resulted in reduced CO₂ elimination compared to ECCO₂R without CRRT at low extracorporeal blood flow rates. In contrast, compared with 25 mmol/L, a low bicarbonate concentration of 16 mmol/L in the substitution solution significantly increased CO₂ elimination, although the difference in patients did not reach statistical significance, which is likely due to the variation in blood flow. Unlike animal experiments, the blood flow in patients was 293 ± 59 ml/min, which was not a fixed value. It is possible that a larger sample size would have enabled the detection of a statistically significant difference.

The main goal of ECCO_2_R in ARDS is to facilitate lung protective ventilation, while avoiding hypercapnic acidosis, additionally, the lung-protective benefits of ECCO_2_R increase with higher device performance. Although ECCO₂R-CRRT systems have demonstrated feasibility and efficacy in managing patients with both hypercapnia and severe kidney injury [2, 14], the bicarbonate concentration in the substitution solution may influence CO₂ elimination by affecting the external bicarbonate load. In the present study, a low bicarbonate concentration of 16 mmol/L in the substitution solution was selected based on animal experiments by Cove et al., which demonstrated effective CO₂ removal with low bicarbonate dialysis [15]. A normal bicarbonate concentration of 25 mmol/L was chosen to reflect the physiological serum plasma range. Our results indicate that a relatively lower bicarbonate concentrations in the substitution solution may facilitate CO₂ elimination and provided a acceptable physiological base. Additionally, both for animals and patients of hypercapnic acidosis, a good kidney function favors less tendency towards metabolic acidosis. This finding is consistent with previous in vitro studies showing more efficient CO₂ removal with lower bicarbonate levels in the substitution fluid [9]. Importantly, this study provides, for the first time, evidence supporting the feasibility and reliability of using an ECCO₂R-CRRT system with a low bicarbonate concentration substitution solution in both animal models and clinical settings.

A bicarbonate-rich substitution solution may counteract the overall effectiveness of CO₂ removal by increasing the CO₂ load in the extracorporeal blood with CO₂. Additionally, due to the low efficiency at the blood flow of 200 ml/min, the increased CO_2_ load of normal HCO_3_^−^ group can not be efficiently cleared by the membrane lung and result in the lower VCO_2_ of combined system. The bicarbonate substitution solution, which enters the extracorporeal circuit in the way of pre-dilution, dilutes bicarbonate concentration, meanwhile increases the CO_2_ load prior to membrane lung. Compared with normal bicarbonate, the lower bicarbonate substitution solution enhanced CO_2_ removal of the combined system. But this increase is relative rather than absolute. The effect is the relative increase of CO_2_ removal due to reduced CO_2_ load. A pilot study by John et al. in patients with hypercapnic acidosis found that CO₂ elimination with the combined ECCO₂R-CRRT systems was about 5% lower than with ECCO₂R alone [8]. Using a low bicarbonate substitution solution, as demonstrated in this study, is a potential solution to conducive to CO₂ removal, while there was no metabolic acidosis ocurred in both hypercapnic animals and ARDS patients. For patients, the next day of intervention during ECCO_2_R, the arterial pH was 7.44 ± 0.02, PaCO_2_ was 42.1 ± 3.5 mmHg, HCO_3_^−^ was 25.0 ± 4.5 mmol/L, cl^−^ was 102.0 ± 4.3 mmol/L. Noteworthy, with appropriate caution regarding renal compensation and metabolic acidosis, a low bicarbonate concentration in the substitution solution may be beneficial for patients with normal renal function, respiratory acidosis, and low extracorporeal blood flow rates. In this setting, the reduced CO_2_ load associated with a lower bicarbonate concentration may result in higher CO_2_ removal efficiency. Conversely, in patients with severe renal failure and metabolic acidosis, a normal bicarbonate concentration is more appropriate. At a minimum, the use of a low bicarbonate concentration should be delayed until metabolic acidosis is corrected and renal compensation is restored. The optimal timing for initiating a low bicarbonate concentration therefore warrants further investigation. Additionally, future studies should emphasize electrolyte disturbances, which necessitate close monitoring and meticulous adjustment of the CRRT prescription.

Another approach is to increase blood flow, which is a key determinant of VCO₂ in ECCO₂R. Our findings indicate that at the blood flow of 350 mL/min, the high efficiency of the membrane lung can compensate for the additional CO₂ introduced by a bicarbonate-rich substitution solution. It remains unclear whether non-bicarbonate-based substitution solutions offer a greater CO₂ removal effect. However, lactate-based solutions may not be suitable for critically ill patients with multiple organ failure. Additionally, citrate-based solutions, which can provide effective anticoagulation, are currently unavailable at the studied blood flow of 350 mL/min due to the high citrate dosing requirements.

The average VCO₂ in ARDS patients reached 70 mL/min in this study, which was higher than that reported in previous studies [2, 8]. This might be partially attributable to the total surface area of the membrane lung used in our study. The SUPERNOVA study [3, 16] indicates that ECCO₂R systems with larger membranes (1.30 m^2^) are more effective in CO₂ removal and enabling ultraprotective ventilation than those with smaller membranes (0.59 m^2^). In this study, a 1.81 m^2^ membrane further improved CO₂ clearance. Additionally, the configuration of the ECCO₂R-CRRT system can impact its efficiency. Allardet-Servent et al. found that placing the membrane oxygenator upstream of the hemofilter increased blood flow and CO₂ clearance compared to a downstream position, with other parameters such as cannula size held constant [17]. In this study, the system configuration positions the membrane lung upstream of the hemofilter, which may further improve its effectiveness.

The present study has several limitations. First, due to the configuration of the combined system, it was not possible to collect blood samples at the junction between the membrane lung and the hemofilter. As a result, the CO₂ content at this critical point remains unknown, preventing us from separately calculating the CO₂ removal attributable to CRRT and ECCO_2_R. Second, the study design involved pre-selecting two specific bicarbonate concentrations for the substitution solution. This approach did not account for potential therapeutic variability among individual patients during CRRT, thereby limiting our ability to identify the optimal bicarbonate concentration to maximize CO₂ removal efficiency. Third, the CO_2_ production did not be measured, and the observation period of 30 min may be not long enough to evaluate acid–base balance. Forth, the CRRT settings in this study involved matching the ultrafiltration rate to the total volume of substitution fluid infused. While this approach provided a controlled environment for analysis, it may limit the generalizability of our findings to clinical scenarios involving net fluid loss. Additionally, we did not assess the efficiency of the combined system under alternative CRRT modalities, such as dialysis or post-dilution. These modes introduce additional complexity in calculating CO₂ content within the circuit and may yield different outcomes. Nevertheless, we must emphasize that our results are very preliminary and hypothesis-generating. The impact of bicarbonate concentration on CO_2_ removal and acid–base balance need to be clarified in long-term experiments with more CRRT modes.

## Conclusion

This study, involving hypercapnic animal models and ARDS patients, demonstrates that a low bicarbonate concentration of 16 mmol/L in the substitution solution may be conducive to CO₂ removal in the ECCO₂R-CRRT system, especially at lower blood flow rates. Further research is required to optimize the management of this combined system.

## Supplementary Information

Additional file 1. Table S1. Composition of the substitution solution. Table S2. Electrolyte concentration of the substitution solution. Table S3. Variations of hemodynamic parameters among extracorporeal CO_2_ removal strategies in hypercapnic pigs (n=12).

## Data Availability

The datasets used and/or analyzed in the current study are available from the corresponding author on reasonable request.

## Abbreviations

ARDS: Acute respiratory distress syndrome
CO_2_: Carbon dioxide
CRRT: Continuous renal replacement therapy
CVVH: Continuous veno-venous hemofiltration
ECCO_2_R: Extracorporeal carbon dioxide removal
ECBF: Extracorporeal blood flow
VCO_2_: Carbon dioxide removal rate
FiO_2_: Fraction of inspired oxygen
PEEP: Positive end-expiratory pressure
PaCO_2_: Partial pressure of arterial carbon dioxide
PaO_2_: Partial pressure of arterial oxygen
MAP: Mean arterial pressure
