# Supplementary material for: Effect of low bicarbonate substitution solution on CO2 removal rate in the combined system of extracorporeal CO2 removal and continuous renal replacement therapy
Source: Intensive Care Med Exp. 2025 Nov 19;13:116. doi: 10.1186/s40635-025-00827-8 (PMC12627315; doi:10.1186/s40635-025-00827-8)
Supplement: Supplementary file 1 — Additional file 1. Table S1. Composition of the substitution solution. Table S2. Electrolyte concentration of the substitution solution. Table S3. Variations of hemodynamic parameters among extracorporeal CO2 removal strategies in hypercapnic pigs (n=12). [file 40635_2025_827_MOESM1_ESM.docx]

**Supplementary material**

# Effect of low bicarbonate substitution solution on CO_2_ removal rate in the combined system of extracorporeal CO_2_ removal and continuous renal replacement therapy

Authors: Zhicheng Qian^1,2✝^, Rui Zhang^1✝^, Yuxuan Wang^1,3^, Hao He^1,4^, Shike Geng^1^, Yang Li^1^, Xueyan Yuan^1^,Yi Yang^1^, Haibo Qiu^1^, Songqiao Liu^1,5^, Ling Liu^1*^

^1^Jiangsu Provincial Key Laboratory of Critical Care Medicine, Department of Critical Care Medicine, Zhongda Hospital, School of Medicine, Southeast University, Nanjing, China.

^2^Department of Critical Care Medicine, Beijing Anzhen Nanchong Hospital，Capital Medical University & Nanchong Central Hospital, Nanchong, Sichuan, China

^3^Department of Emergency, Central Hospital Affiliated to Shandong First Medical University, Shandong First Medical University & Shandong Academy of Medical Sciences, Jinan, China.

^4^Department of Critical Care Medicine, West China Hospital/West China School of Medicine, Sichuan University,Chengdu, Sichuan, China.

^5^The First People's Hospital of Lianyungang, The Affiliated Lianyungang Hospital of Xuzhou Medical University, Lianyungang 222000, Jiangsu, China.

^✝^These authors contributed equally

^*^Correspondence to: Ling Liu, liulingdoctor@126.com, Department of Critical Care Medicine, Zhongda Hospital, School of Medicine, Southeast University, Jiangsu Provincial Key Laboratory of Critical Care Medicine. No. 78 Dingjiaqiao Road, Gulou District, Nanjing, Jiangsu, 210009, China.

**Supplementary methods for animals**

Anxiolytic premedication was administered via intramuscular injection of 2 mg/kg esketamine and 0.5 mg/kg midazolam in a stress-free environment. Venous access was obtained via an ear vein using a 20 G indwelling cannula. Anaesthesia was induced using 1–3 µg/kg fentanyl and 2.0–2.5 mg/kg propofol. After achieving an adequate depth of anaesthesia, an endotracheal tube (7.5 or 8.0 mm) was inserted. Anaesthesia was maintained by continuous infusion of midazolam (0.1–0.5 mg/kg/h), propofol (5–10 mg/kg/h), and fentanyl (0.5–1.0 µg/kg/h). Body temperature was maintained throughout the experiment using a heating blanket when necessary. An electrolyte solution (compound sodium chloride; Aodong, China) was continuously infused at 2–3 mL/kg/h. Colloid solution (hydroxyethyl starch) and norepinephrine were administered as needed to maintain a mean arterial pressure (MAP) above 60 mmHg.

A 7 Fr central venous catheter (Arrowg+ard Blue®, ARROW, USA) was inserted into the right femoral vein using the ultrasound-guided Seldinger technique for infusion and blood sampling. Cardiac output was recorded using a PiCCO™ thermodilution catheter. A 14 Fr double-lumen catheter (Arrowg+ard Blue®, ARROW, USA) was inserted into the right jugular vein using the ultrasound-guided Seldinger technique for ECCO₂R.

Following endotracheal intubation, mechanical ventilation (Servo-I, Maquet, Sweden) was initiated in volume-controlled mode with a tidal volume of 6 mL/kg, a respiratory rate of 15 breaths/min, a positive end-expiratory pressure (PEEP) of 5 cmH₂O, and an FiO₂ of 0.5. Hypercapnia was induced by administering 1–1.5 mg/kg/h rocuronium bromide for paralysis and reducing the respiratory rate. To elevate the target PaCO_2_ of 70–89 mmHg, the respiratory rate was reduced and the tidal volume was maintained, titrated according to end-tidal carbon dioxide pressure until constant PaCO_2_, which was confirmed by blood gas analysis. This modeling phase lasted 30 minutes.

During ECCO₂R, anticoagulation was achieved by continuous infusion of unfractionated heparin to maintain an activated clotting time of approximately 300 seconds, due to the higher coagulability of pigs compared to humans (1, 2). The sweep gas flow rate was set at 10 L/min. For CVVH management, 5% sodium bicarbonate was added to the basic replacement fluid dilutes (Shijiazhuang Fourth Pharmaceutical Company, China) under sterile conditions to achieve target bicarbonate concentrations of 25 and 16 mmol/L. A 10% calcium gluconate solution was infused via the central venous cannula. The detailed compositions of the replacement fluid dilutes are presented in Table S1 and Table S2. The CVVH effluent flow rate was set at 30 mL/kg/h with 100% pre-dilution.

**Experimental protocol for pigs**

Six therapeutic strategies as follows were implemented in randomized sequence:

1. ECCO_2_R alone with an ECBF of 200 mL/min;
2. ECCO_2_R with an ECBF of 200 mL/min combined CVVH at a bicarbonate concentration of 16 mmol/L in the replacement fluid dilutes;
3. ECCO_2_R with an ECBF of 200 mL/min combined CVVH at a bicarbonate concentration of 25 mmol/L in the replacement fluid dilutes;
4. ECCO_2_R alone with an ECBF of 350 mL/min;
5. ECCO_2_R with an ECBF of 350 mL/min combined CVVH at a bicarbonate concentration of 16 mmol/L in the replacement fluid dilutes;
6. ECCO_2_R with an ECBF of 350 mL/min combined CVVH at a bicarbonate concentration of 25 mmol/L in the replacement fluid dilutes;

Each therapy maintained for 30 minutes with 15-minute washout period to minimize the carry-over effects. Both ECCO_2_R and CRRT were halted during the washout period. If necessary, the respiratory rate was adjusted to stabilized the PaCO_2_ within 70 to 89 mmHg before the next therapy. During each step, the ventilatory settings remained unchanged.

**Supplementary protocol for ARDS patients**

After the stabilization of respiratory and hemodynamic conditions, three strategies (ECCO_2_R alone, ECCO_2_R-CVVH with HCO_3_^-^ of 16 mmol/L, ECCO_2_R-CVVH with HCO_3_^-^ of 25 mmol/L) were implemented in random order. Each step maintained for 30 minutes, without a washout period between three strategies, to prevent repeated increases in PaCO_2_. The study should be stopped when the patient met any of the following criteria: (1) hemodynamic instability with MAP dropped by 20% or systolic blood pressure < 90 mmHg, or severe arrhythmias; (2) hypoxemia with SpO_2_ < 90% for more than 2 minutes, despite increment of FiO_2_. The initial ventilator parameters, effluent flow rate of CVVH, sweep gas flow and ECBF were set by the attending physician.

**Respiratory mechanics data computation**

The measurement of respiratory mechanics was conducted by inspiratory and expiratory hold for 2-3 seconds, under the condition of volume-controlled mode and no spontaneous breathing effort. Driving pressure (DP) was calculated as the difference between plateau pressure (Pplat) and total PEEP(3). Respiratory system compliance was calculated as the ratio between VT and DP.

**Monitoring of adverse events and survival**

The adverse event (AE) was defined as: study related when it could be attributed to a study procedure and could readily have been produced by the study procedure; or non-study related when it was related primarily to the underlying disease or to ARDS and its sequelae. After initiation of ECCO_2_R, patients were monitored for AEs until hospital discharge or day 8 post enrollment, whichever occurred first(4). Survival during hospitalization was also recorded.

**Reference**

1. Mizuno T, Tsukiya T, Takewa Y, Tatsumi E. Differences in clotting parameters between species for preclinical large animal studies of cardiovascular devices. Journal of artificial organs : the official journal of the Japanese Society for Artificial Organs. 2018;21(2):138-41.

2. Siller-Matula JM, Plasenzotti R, Spiel A, Quehenberger P, Jilma B. Interspecies differences in coagulation profile. Thrombosis and haemostasis. 2008;100(3):397-404.

3. Amato MB, Meade MO, Slutsky AS, Brochard L, Costa EL, Schoenfeld DA, et al. Driving pressure and survival in the acute respiratory distress syndrome. The New England journal of medicine. 2015;372(8):747-55.

4. Chittamma A, Vanavanan S. Comparative study of calculated and measured total carbon dioxide. Clin Chem Lab Med. 2008;46(1):15-7.

Table S1. Composition of the substitution solution.

| HCO_3_^-^ (mmol/L) | Total volume (mL) | Basic solution (mL) | 5% NaHCO_3_ (mL) | 10% KCl (mL) |
| --- | --- | --- | --- | --- |
| 25 | 4180 | 4000 | 168 | 12 |
| 16 | 4119 | 4000 | 107 | 12 |

Table S2. Electrolyte concentration of the substitution solution.

| HCO_3_^-^ (mmol/L) | Na^+^ (mmol/L) | K^+^ (mmol/L) | Cl^-^ (mmol/L) | Mg^2+^ (mmol/L) | Glucose (mmol/L) |
| --- | --- | --- | --- | --- | --- |
| 25 | 141 | 4.0 | 110 | 0.75 | 10 |
| 16 | 140 | 4.0 | 110 | 0.75 | 10 |

| Table S3. Variations of hemodynamic parameters among extracorporeal CO_2_ removal strategies in hypercapnic pigs (n=12).   \| Variables \| Before modeling \| Control group \| \| \| Normal HCO_3_^-^group \| \| \| Low HCO_3_^-^group \| \| \| \| --- \| --- \| --- \| --- \| --- \| --- \| --- \| --- \| --- \| --- \| --- \| \| Baseline \| After removal \| *P* value \| Baseline \| After removal \| *P* value \| Baseline \| After removal \| *P* value \| \| Hemodynamics at an ECFB of 200 mL/min \| \| \| \| \| \| \| \| \| \| \| \| Heart rate (bpm) \| 110 ± 13 \| 127 ± 8 \| 123 ± 9 \| 0.367 \| 125 ± 9 \| 123 ± 6 \| 0.752 \| 125 ± 9 \| 123 ± 6 \| 0.908 \| \| MAP (mmHg) \| 109 ± 13 \| 103 ± 11 \| 101 ± 9 \| 0.149 \| 103 ± 9 \| 102 ± 8 \| 0.764 \| 103 ± 9 \| 102 ± 8 \| 0.946 \| \| CO (L/min) \| 4.8 ± 0.5 \| 5.3 ± 0.5 \| 5.2 ± 0.7 \| 0.515 \| 5.3 ± 0.7 \| 5.2 ± 0.5 \| 0.883 \| 5.3 ± 0.7 \| 5.2 ± 0.5 \| 0.930 \| \| Hemodynamics at an ECFB of 350 mL/min \| \| \| \| \| \| \| \| \| \| \| \| Heart rate (bpm) \| 110 ± 24 \| 125 ± 36 \| 118 ± 21 \| 0.002 \| 120 ± 31 \| 116 ± 19 \| 0.021 \| 119 ± 33 \| 115 ± 16 \| 0.027 \| \| MAP (mmHg) \| 110 ± 31 \| 105 ± 22 \| 102 ± 18 \| 0.173 \| 98 ± 21 \| 97 ± 15 \| 0.165 \| 95 ± 21 \| 97 ± 13 \| 0.136 \| \| CO (L/min) \| 4.8 ± 1.3 \| 5.2 ± 1.6 \| 5.0 ±0 .5 \| 0.315 \| 5.5 ± 1.6 \| 5.2 ± 0.6 \| 0.412 \| 5.3 ± 1.2 \| 5.5 ± 1.3 \| 0.246 \| |
| --- | --- | --- | --- | --- | --- | --- | --- | --- | --- | --- | --- | --- | --- | --- | --- | --- | --- | --- | --- | --- | --- | --- | --- | --- | --- | --- | --- | --- | --- | --- | --- | --- | --- | --- | --- | --- | --- | --- | --- | --- | --- | --- | --- | --- | --- | --- | --- | --- | --- | --- | --- | --- | --- | --- | --- | --- | --- | --- | --- | --- | --- | --- | --- | --- | --- | --- | --- | --- | --- | --- | --- | --- | --- | --- | --- | --- | --- | --- | --- | --- | --- | --- | --- | --- | --- | --- | --- | --- | --- | --- | --- | --- | --- | --- | --- | --- | --- | --- | --- | --- | --- | --- | --- | --- | --- | --- | --- | --- |

ECFB, extracorporeal blood flow; MAP, mean arterial pressure; CO, cardiac output.

*P* value denotes comparisons between baseline and after removal at the same treatment strategy.
